# Hospital Nurses’ Perception of Death and Self-Reported Performance of End-of-Life Care: Mediating Role of Attitude towards End-of-Life Care

**DOI:** 10.3390/healthcare8020142

**Published:** 2020-05-22

**Authors:** Hyo-Jin Park, Yun-Mi Lee, Mi Hwa Won, Sung-Jun Lim, Youn-Jung Son

**Affiliations:** 1 Department of Nursing, Kyungnam College of Information & Technology, Busan 47011, Korea; parkhj@eagle.kit.ac.kr; 2 Department of Nursing, College of Medicine, Inje University, Busan 47392, Korea; lym312@inje.ac.kr; 3 Department of Nursing, Wonkwang University, Iksan, Jeonbuk 54538, Korea; mihwon7729@gmail.com; 4 Red Cross College of Nursing, Chung-Ang University, 84 Heukseok-ro, Dongjak-Gu, Seoul 06974, Korea; seoungjunelim@gmail.com

**Keywords:** attitude, end-of-life care, death, hospital, nurses, perceptions

## Abstract

Few studies have explored how nurses in acute care hospitals perceive and perform end-of-life care in Korea. Therefore, this study aimed to evaluate the influence of nurses’ perceptions of death on end-of-life care performance and analyze the mediating role of attitude towards end-of-life care among hospital nurses. This cross-sectional study included a total of 250 nurses who have had experience with end-of-life care from four general hospitals in Korea. We used the Korean validated tools with the View of Life and Death Scale, the Frommelt Attitudes Toward Care of the Dying (FATCOD) scale, and the performance of end-of-life care. Hierarchical linear regression and mediation analysis, applying the bootstrapping method. The results of hierarchical linear regression showed that nurses’ positive perceptions of death and attitude towards end-of-life care were significantly associated with their performance of end-of-life care. A mediation analysis further revealed that nurses’ attitude towards end-of-life care mediates the relationship between the perceptions of death and performance of end-of-life care. Our findings suggest that supportive and practical death educational programs should be designed, based on nurses’ professional experience and work environment, which will enable them to provide better end-of-life care.

## 1. Introduction

Palliative care involves treatment of individuals who have life-threatening illnesses, in which a cure or complete reversal of the disease and its process is no longer possible [1,2,3,4]. Patients facing unbearable suffering from terminal illnesses experience multiple physical, psychosocial, and cognitive processing problems at the end-of-life stage [3,4]. As part and parcel of palliative care, end-of-life (EOL) care involves care being given in the last part of a patient’s life, typically in the last few months, depending on the primary diagnosis and clinical course [5]. Global interest in EOL care is growing, for compelling demographic and epidemiological reasons [1,2]. The population of the world is ageing and the number of people dying each year is set to rise [1,6]. In South Korea, the population of older adults is expected to grow dramatically, due to significant increases in life expectancy and population aging [4]. Thus, delivering high quality of end-of-life (EOL) care to dying patients has long been prioritized in national and international research agendas [7,8,9]. Acute care hospitals play an important role in managing patients with life-limiting diseases and providing EOL care [5,9]. Unfortunately, a recent review revealed that many hospitals have failed to sufficiently manage symptom distress of dying patients [3]. Inadequate EOL care in hospitals has resulted in many unresolved complaints [4,8]. Moreover, hospitals may be poorly equipped to reliably provide proficient EOL care [5,8].

Hospital nurses in acute care hospitals are frequently exposed to dying patients and death in their workplace [3,8]. Notwithstanding that a patient’s death is one of the most common stressors in clinical settings, nurses are unlikely to express their feelings and evaluations about such occurrences with their colleagues [10,11,12]. Prior studies have reported that nurses had feelings of inadequacy, helplessness, or distress in supporting dying patients [12,13]. In addition, nurses were uncertain how to behave in the context of a patient’s death [9]. This might be based on the concept of death, and responses to death vary across cultural backgrounds, religious beliefs, social values, and traditions [13,14,15]. 

Perception is an individual’s view and unique sentiments that incorporate an individual’s own memories and experiences in the process of understanding a situation, making it a powerful driving force for action [16]. In this regard, nurses with negative perceptions of death are more likely to experience limitations in supportive EOL care and would suffer from lack of knowledge and adequate training [17]. On the contrary, nurses with positive perceptions of death will have less fear of death or death anxiety [18]. Therefore, nurses need to recognize and confront their own perceptions of death. Importantly, encouraging nurses to think about their views on their own death may be a good coping mechanism and may enable them to deliver better EOL care [18,19]. However, evidence on this issue proved to be inconsistent, and thus, brought about a need for reinvestigation. Previous studies have reported that nurses’ negative attitudes towards EOL care may be related with poor quality of EOL care [4,11]. A better understanding of the impact of attitude on the performance of EOL care would enable us to overcome barriers and optimize EOL care [15]. Furthermore, it is important for hospital nurses to deliver better EOL care for patients considering the wider aspects care such as physical, psychosocial, and spiritual aspects of patient care [5].

Based on the existing literature [1,2,3,4,5,6,7,8], Western and non-Western countries are found in different views: Religious and spiritual expectations surrounding death; laws and opinions about euthanasia. In addition, the practices and beliefs of healthcare professionals of EOL care are also framed by culture [1,2]. In 2018, Korea legislated the Well-dying Act [4]. Accordingly, EOL care in Korea is still not actively used. More importantly, there is limited data regarding the relationships between nurses’ perceptions, attitude toward EOL care, and performance for dying patents in Korea [20,21]. Thus, the purpose of the present study was to examine the impact of perceptions of death on EOL care performance among hospital nurses. Furthermore, we aimed to identify the mediating role of attitude towards EOL care on the relationship between perceptions of death and self-reported performance of EOL care.

## 2. Materials and Methods 

### 2.1. Study Design and Participants

This study adopted a descriptive cross-sectional research design using convenience sampling on a total of 250 nurses who were recruited from four general hospitals with over 300 beds in Pusan, South Korea. The inclusion criteria involved full-time registered nurses who have had experience with EOL care and actively conduct bedside patient care. The exclusion criteria involved newly-employed nurses who were trainees under the supervision of preceptors or nurse managers. The sample size was estimated by using G*power version 3.1 [22]. A sample size of 215 participants was required for a multiple linear regression to detect a moderate effect size (f^2^=0.10) with an alpha of 0.05, and a power level of 0.90 using ten predictors. At the beginning, 250 participants took part in this study based on a 15% potential dropout rate. Our final data included a total of 230 participants, excluding 20 insufficiently answered questionnaires.

### 2.2. Instruments

#### 2.2.1. Socio-Demographic and Clinical Characteristics 

Socio-demographic characteristics (age, educational level, marital status, total years of practice, position title, type of nursing unit such as general ward, intensive care unit (ICU), and palliative care unit, experiences of EOL care education, and experiences of losing an acquaintance) were collected using a self-reported questionnaire.

#### 2.2.2. Perceptions of Death

We adopted the Korean version of the View of Life and Death Scale [23] to measure perceptions of death. This scale was designed to measure participants’ views of life and death. This validated scale consists of 57 items, including subscales concerning the meaning of death, death concerns, and life respect will. For each item, the respondent would tick a box from a 1-to-7-point Likert scale, with 1 being not at all true, 4 being somewhat true, and 7 being very true. The summary score ranged from 57-399, with higher scores indicating higher levels of perceptions of death. The Cronbach’s alpha coefficient in this study was 0.864.

#### 2.2.3. Attitude towards EOL Care

We used the Korean version of The Frommelt Attitudes Toward Care of the Dying (FATCOD) scale [24]. Frommelt (1991) developed the original FATCOD scale to assess individuals’ attitudes toward providing care to dying patients [25]. The scale consists of 30-items, including 15 positively and negatively worded items each [24]. The scale uses a four point Likert scale ranging from 1 to 4, and its total score ranges from 30 to 120. Higher scores indicate more positive attitudes toward providing care for dying patients [24]. The Cronbach’s alpha coefficient was 0.860 in this study.

#### 2.2.4. Self-Reported Performance of EOL Care

The performance of EOL care was measured using the terminal care performance scale developed by Park and Choi in Korea [26]. It was used to evaluate Korean nurses EOL care performance. Its validity has been established [26]. It assessed the level of EOL care performance of nurses, including physical, psychological, and spiritual domains. The scale consists of 22 items rated on a 1 (not at all true)-to 4 (completely true) point Likert scale. The summary score ranged from 22-88. Higher scores indicate higher level of performance of EOL care [26]. The Cronbach’s alpha coefficient in this study was 0.911.

### 2.3. Ethical Consideration and Data Collection

The institutional research board in the first authors’ institution approved this study protocol (No. 2-1041024-AB-N-01-20160219-HR-361). This study was conducted in accordance with the Declaration of Helsinki. Data were collected after obtaining permission from the chief of nursing administrator of each hospital from September 1 to 20, 2016. Two trained research assistants visited each unit at around their shift change to explain the purpose, method, and ethics of the study, before giving out questionnaire to those who voluntarily agreed to participate in it. All eligible nurses received an information sheet with details of the study, their rights regarding participation in the study and withdrawal. The completed questionnaires were sealed in envelopes before collection to ensure the anonymity and confidentiality of the participants. All information collected about the participants was kept strictly confidential. Only the research team could access the encrypted data file. All hard copies of the surveys were kept in a locked cabinet and will be destroyed within five years after the completion of the study.

### 2.4. Data Analysis

All data were analyzed using IBM SPSS Statistics version 23 (SPSS Inc., Chicago, IL, USA). Descriptive statistics (mean ± standard deviations) and calculated for continuous variables. The frequencies and percentages were calculated for categorical variables. The independent t-test, one-way analysis of variance, and the post hoc test (Duncan’s multiple comparison test) were performed to test for the mean differences between groups. Pearson’s correlation coefficient was used to test the relationship between variables.

Hierarchical linear regression analysis was conducted to identify the impact of perceptions of death and attitude and self-reported performance of EOL care after adjusting for covariates. Additionally, the potential mediating effect of attitude towards EOL care was examined by performing Baron and Kenny’s analysis technique [27] and by subsequently applying the bootstrapping method to obtain confidence intervals (CIs) based on 5,000 resamples [28]. A value of *p* < 0.05 was considered statistically significant.

## 3. Results

### 3.1. Sample Characteristics

As shown in Table 1, all the participants in our study were female, with a mean age of 31.4 (SD 8.43) years old. Approximately half (57.8%) of the participants were university graduates and 73.5% lived alone. Average years of clinical practice was approximately 6.84 (SD 6.10) years. The majority (82.2%) of participants were staff nurses and 43.5% of the respondents worked in the general medical and surgical units. The majority of participants had experience with both EOL care education (61.7%) and losing an acquaintance (82.6%).

### 3.2. The Level and Correlations Among Perceptions of Death, Attitude, and Self-Reported Performance of EOL Care

The mean (SD) scores of perceptions of death, attitude, and performance of EOL care were 255.24 (29.78), 90.51 (10.27), and 54.46 (8.99), respectively (Table 2). Perceptions of death was significantly correlated with attitude (r = 0.47, *p* < 0.001) and performance of EOL care (r = 0.49, *p* < 0.001). Attitude towards EOL care was also significantly correlated with performance of EOL care (r = 0.57, *p* < 0.001) (Table 2). 

### 3.3. Difference in Performance of EOL Care by Socio-Demographic and Clinical Characteristics

As shown in Table 3, the level of performance of EOL care were higher in older nurses (F = 7.51, *p* = 0.001) and those with longer years of practice (F = 21.16, *p* < 0.001). The level of performance of EOL care in palliative care units was also the highest among the types of nursing units (F = 12.79, *p* < 0.001). There were no differences in the mean scores of performance of EOL care levels based on educational level, marital status, position title, experiences of EOL care education, and experience of losing an acquaintance.

### 3.4. Hierarchical Linear Regression Analysis Predicting Self-reported Performance of EOL Care

In hierarchical linear regression, we first included socio-demographic and clinical characteristics (age, educational level, marital status, total years of practice, position title, type of nursing unit, experiences of EOL care education, and experiences of losing an acquaintance) in Model I. We then added perceptions of death in Model II. Finally, we added attitude towards EOL care in Model III (Table 4). 

Regression analyses showed that both perceptions of death (*β* = 0.23, *p* < 0.001) and attitude towards EOL care (*β* = 0.33, *p* < 0.001) predicted self-reported performance of EOL care. In addition, nurses who had longer years of practices (*β* = 0.22, *p* < 0.018) and worked in specialized care units (*β* = 0.32, *p* < 0.001) were more likely to perform EOL care. The overall model significantly explained 56.1% of the variance in self-reported performance of EOL care (F=28.03, *p* < 0.001). 

### 3.5. Mediating Effect of Nurses’ Attitude With Respect to Perceptions of Death and Performance of EOL Care.

**Figure 1:**
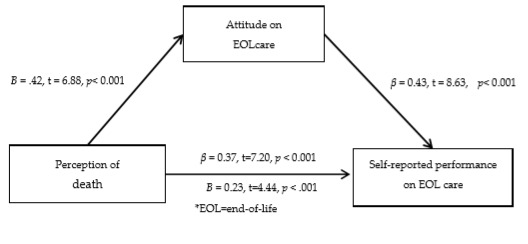
Mediating Role of Attitude on EOL Care on The Relationships Between Perception of Death and Self-reported Performance on EOL Care.

Figure 1 shows the mediating effect of attitude towards EOL care in the relationship between perceptions of death and performance of EOL care. In step 1, the perceptions of death was significantly associated with attitude towards EOL care (*β* = 0.42, *p* < 0.001, 95% CI = 0.09 to 0.17). In step 2, the perceptions of death was significantly associated with performance of EOL care (*β* = 0.37, *p* < 0.001, 95% CI = 0.08 to 0.14). In step 3, attitude towards EOL care was also significantly associated with performance of EOL care (*β* = 0.43, *p* < 0.001, 95% CI = 0.29 to 0.47). 

However, after adding attitude towards EOL care, β from perceptions of death was reduced in relation to the performance of EOL care (*β* = 0.23, p<0.001, 95% CI = 0.04 to 0.10). This result showed that the attitude towards EOL care mediated the relationship between perceptions of death and self-reported performance of EOL care. Bootstrapping analyses demonstrated that the indirect effect was significant (95% CI 0.20 to 0.38).

## 4. Discussion

Caring for dying patients is particularly difficult among nurses because it raises nurses’ negative cognitive bias, including emotional distress, and may lead them to avoid managing dying patients altogether [29,30]. Consequently, it is important to describe how the perceptions of nurses influence their administration of EOL care. 

Our main finding revealed that nurses’ positive perceptions of death, and their attitude towards EOL care, were significantly associated with better performance of EOL care. This result was consistent with previous studies, indicating that the more negative a nurse’s perceptions or attitude towards death, the less likely the he is to deliver EOL care [31,32,33]. Our finding was likewise similar to the study of Hussin et al. [18], which reported that Malaysian nurses’ knowledge and attitude towards EOL care were low, and that these two factors were associated with the perceived quality of EOL care. This is understandable, given that caring for dying patients implies a high level of emotional involvement [34]. According to previous studies, many nurses handling EOL patients may be caught in fear, uncertainty, and emotional exhaustion, which often lead to lapses in providing for emotional support or ignoring patients’ cues [30,33]. Nurses’ good perceptions can be raised by engaging in discussions about fear, anxiety, and belief with nursing colleagues and experienced superiors [15,29,34]. Accordingly, nurses have to be professionally trained on EOL issues; otherwise, they may cause emotional damage not only to their patients, but even to themselves [30].

One Korean study reported that Korean nurses were found to regard caring for dying patients in a negative light [35]. It is this negative emotional response that lead them to avoid dying patients [36]. In comparison to Western countries, Korean people are less likely to discuss death or dying situations, due to the belief that it is a failure on their part, as well as the traditional Korean view of death by a Confucian society, where people tend to extend meaningless life-prolonging treatment [4,35]. For this reason, different cultural backgrounds of nurses can be also considered as a contributing to positive or negative perceptions and attitudes [31,37]. 

In particular, our study found that nurses’ attitudes towards EOL care mediated the relationship between perceptions of death and performance of EOL care. This result was consistent with previous findings, which showed that some nurses found difficulty in establishing close relationships with dying patients and their families due to fear of death, which in turn led to neglect for dying patients’ needs [11,38]. It was also supported by a recent review on perceptions of death among Brazilian health professionals, revealing that health professionals, who think about death as a natural process of life, are more likely to have more positive attitudes towards alleviating patients’ suffering [13,34]. On the other hand, some health professionals may be full of negativity resulting from unpreparedness in supporting the dying process of patients [31,39]. One study showed that positive attitudes towards EOL care for the dying is enhanced through actual caring for dying patients, which contribute to nurses’ personal growth [29]. Greater exposure leads to greater acceptance, which in turn, develops into a more positive overall attitude of nurses [40,41]. It is for this reason that nurses should first be aware of their own values and attitudes towards patients with terminal illnesses. Furthermore, nurses must care for themselves and build resilience and self-confidence to be able to provide optimal EOL care [20,34]. In addition, nurse administrators and hospital policymakers should develop strategies to enhance nurses’ positive perceptions and improve their attitude toward EOL care, through external professional resources, such as professional training and emotional support [15]. Future studies are needed to investigate what misunderstanding or stereotypes influence nurses’ attitudes. 

Additionally, the present study showed that the level of performance of EOL care were significantly lower in nurses who were younger, or had fewer years of experience, as compared to their older or more experienced colleagues. This finding supported the results of previous studies that younger nurses may have stronger fear or psychological burden for death or the dying process and consequently, more negative attitudes toward EOL care [12,15]. The experience of unpredictable and stressful environments might be more uncomfortable to novice nurses who have relatively insufficient education in the area of EOL care [8,41]. Younger nurses may also be less-equipped for dealing with emotional burdens [40,42]. On the contrary, nurses with more professional experience are more likely to have better communication with dying patients [18,34]. In particular, nurses with EOL care-experience in hospital settings help new nurses adjust to EOL care with their excellent nursing skills and through sharing their previous experiences. It would be important to design the depth of death education at the undergraduate level for greater exposure, along with providing more practical experience with dying patients, and creating a mentoring system for younger, inexperienced nurses in the workplace. Not surprisingly, our study also found that nurses who work at ICU and palliative care units were more likely to perform EOL care than those who work in general wards. From the nature of ICU or palliative care units, patients there are more likely to face EOL cases than in other units [42,43].

Our study emphasized that nurses’ performance of EOL care were affected by how they perceive death. Nursing education must include compassionate care or holistic management for hospital nurses, and should focus on reducing negative perceptions and improving positive attitudes toward EOL care. Additional research is needed to explicate the results of this study. 

## 5. Limitations

The participants were recruited from four general hospitals of single region using a convenience sampling Thus, the findings may not be used to generalize the experiences of nurses who care for dying patients in various healthcare settings. We also adopted self-reported questionnaires for identifying nurses’ performance of EOL care. Assessment for actual performance of EOL care may be needed in addition to their perceived performance. Moreover, the performance of EOL care was developed and validated in Korea. Thus, future researches are needed to validate this tool in various cultural contexts and consider the development of the standardized validated tool for measuring EOL care performance. Even though this study was conducted based on the clear protocols for data collection and analysis, the researchers’ religious beliefs or cultural backgrounds may have an influence on the reporting. Finally, the study only included registered nursing staff who experience care for patients at an end-of life stage. Interdisciplinary staff who care for patients on a dying trajectory may also have unique experiences that should be further explored in future studies.

## 6. Conclusions

Our findings provide evidence that nurses’ good perceptions of death influenced nurses’ better performance of EOL care mediated by attitude towards EOL care in acute care hospital settings. To improve hospital nurses’ positive perceptions of death, integrated and continuing training programs related to death and care for dying patients should be developed based on their clinical experiences and work environment. Healthcare facilities and nurse managers alike play a significant role in assisting novice nurses, and nurses who work in general wards or non-palliative care units, have rewarding experience on EOL care through practical educational courses. Furthermore, death and dying as topics should be incorporated as a part of the undergraduate nursing curriculum. 

## Figures and Tables

**Table 1:** Socio-demographic and Clinical Characteristics of Hospital Nurses (N = 230).

Characteristics	n (%)	Mean ± SD
Age (years)		31.44 ± 8.43
22~29	124 (53.9)	
30~39	71 (30.9)	
≥40	35 (15.2)	
Educational level		
College (3 years)	97 (42.2)	
University (4 years)	113 (49.1)	
Graduate school	20 (8.7)	
Marital status		
Single	169 (73.5)	
Married/Partnered	61 (26.5)	
Total years of practice		6.84 ± 6.10
1-4	119 (51.8)	
5-9	47 (20.4)	
≥10	64 (27.8)	
Position title		
Staff nurse	189 (82.2)	
Charge nurse	41 (17.8)	
Type of nursing unit		
Medical & surgical wards	100 (43.5)	
Intensive care unit	54 (23.5)	
Palliative care unit	76 (33.0)	
Experiences of EOL care education		
No	88 (38.3)	
Yes	142 (61.7)	
Experiences of losing an acquaintance		
No	40 (17.4)	
Yes	190 (82.6)	

EOL = end-of-life.

**Table 2:** Descriptive Statistics and Correlations Among Perceptions of Death, Attitude and Self-reported Performance on EOL Care of Hospital Nurses. (*N* = 230).

Variables	Mean ± SD	Min	Max	Possible Range	Correlation Coefficients	
					1	2
					r (*p*)	r (*p*)
1. Perceptions of death	255.24 ± 29.78	145	356	57–399		
2. Attitude on EOL care	90.51 ± 10.16	37	115	30–120	0.47 (< 0.001)	
3. Performance on EOL care	54.46 ± 8.99	30	86	22–88	0.49 (< 0.001)	0.57 (< 0.001)

EOL = end-of-life.

**Table 3:** Differences in Performance on EOL Care by Socio-demographic and Clinical Characteristics of Hospital Nurses. (*N* = 230).

Characteristics	Mean ± SD	t or F	*p*
Age (years) *			
22~29^a^	52.48 ± 8.08	7.51	0.001
30~39^b^	56.11 ± 9.32		a < b,c
≥ 40^c^	58.11 ± 9.80		
Educational level			
College (3 years)	54.27 ± 8.92	0.07	0.931
University (4 years)	54.68 ± 9.14		
Graduate school	54.10 ± 8.97		
Marital status			
Single	55.20 ± 8.617	1.97	0.052
Married/Partnered	52.41 ± 9.75		
Total years of practice*			
1–4^a^	51.18 ± 7.66	21.16	<0.001
5–9^b^	56.19 ± 9.96		a < b < c
≥10^c^	59.28 ± 8.09		
Position title			
Staff nurse	54.28 ± 8.59	−0.64	0.583
Charge nurse	55.27 ± 10.73		
Type of nursing unit*			
Medical & surgical units^a^	51.59 ± 7.06	12.79	<0.001
Intensive care unit^b^	54.59 ± 11.61		a < b < c
Palliative care unit^c^	58.23 ± 7.68		
Experiences of EOL care education			
No	53.57 ± 8.86	1.18	0.237
Yes	55.01 ± 9.06		
Experiences of losing an acquaintance			
No	53.58 ± 8.51	−0.681	0.479
Yes	54.64 ± 9.10		

EOL = end-of-life. * The same symbols indicate significant difference among groups (a, b and c) based on Duncan test for multiple comparison.

**Table 4:** Hierarchical Linear Regression Analysis Predicting Self-reported Performance on EOL Care among Hospital Nurses. (*N*=230).

Variables	Step 1				Step 2				Step 3			
		95% CI				95% CI				95% CI		
	*β*	Low	High	t *(p)*	*β*	Low	High	t *(p)*	*β*	Low	High	t *(p)*
Socio-demographic and clinical characteristics												
Age	0.01	−0.12	0.34	0.97 (0.332)	0.01	−0.21	0.22	0.05 (0.960)	−0.02	−0.22	0.17	−0.21 (0.832)
Educational level (1 = above university)	0.08	−0.62	3.45	1.39 (0.175)	0.05	−0.89	2.75	1.01 (0.313)	0.07	−0.43	2.93	1.47 (0.143)
Marital status (1 = married/partnered)	−0.09	−4.38	0.51	−1.56 (0.120)	−0.06	−3.46	0.97	−1.10 (0.271)	−0.03	−2.74	1.36	−0.66 (0.510)
Total years of practice	−0.18	−0.05	0.59	1.66 (0.099)	0.21	0.003	0.61	2.16 (0.033)	0.22	−0.06	0.59	2.38 (0.018)
Position title (1 = charge nurse)	0.10	−0.70	5.31	1.51 (0.137)	0.07	−0.99	4.44	0.25 (0.213)	0.06	−1.19	3.83	1.03 (0.301)
Type of nursing unit (1= specialized care unit *)	0.46	6.19	10.42	7.75 (<0.001)	0.39	5.08	8.96	7.14 (<0.001)	0.32	3.97	7.62	6.25 (<0.001)
Experiences of EOLC education (1 = yes)	−0.06	−4.29	1.12	−1.16 (0.249)	−0.02	−2.85	2.07	−0.31 (0.755)	−0.01	−2.36	2.18	−0.08 (0.939)
Experiences of losing an acquaintance (1 = yes)	−0.04	−2.76	1.48	−0.59 (0.554)	−0.01	−2.10	1.73	−0.19 (0.849)	−0.03	−2.26	1.29	−0.54 (0.590)
Perceptions of death					0.37	0.08	0.14	7.20 (<0.001)	0.23	0.04	0.10	4.44 (<0.001)
Attitude on EOL care									0.33	0.20	0.39	6.29 (<0.001)
R², Adjusted R², F (*p*), △R	0.36, 0.33, 15.55 (< 0.001), 36				0.48, 0.46, 22.77 (<0.001), 0.12				0.56, 0.541, 28.03 (<0.001), 0.08			

EOL = end-of-life. * Intensive care unit & palliative care unit.
